# Machine learning to predict risk for community-onset *Staphylococcus aureus* infections in children living in southeastern United States

**DOI:** 10.1371/journal.pone.0290375

**Published:** 2023-09-01

**Authors:** Xiting Lin, Ruijin Geng, Kurt Menke, Mike Edelson, Fengxia Yan, Traci Leong, George S. Rust, Lance A. Waller, Erica L. Johnson, Lilly Cheng Immergluck

**Affiliations:** 1 Morehouse School of Medicine, Department of Microbiology/Biochemistry/Immunology and Clinical Research Center, Atlanta, Georgia, United States of America; 2 Septima, Copenhagen, Denmark; 3 InterDev, Roswell, Georgia, United States of America; 4 Morehouse School of Medicine, Department of Community Health and Preventive Medicine, Atlanta, Georgia, United States of America; 5 Emory University, Rollins School of Public Health, Department of Biostatistics & Bioinformatics, Atlanta, Georgia, United States of America; 6 College of Medicine, and Center for Medicine and Public Health, Florida State University, Tallahassee, Florida, United States of America; Johns Hopkins University, UNITED STATES

## Abstract

*Staphylococcus aureus* (*S*. *aureus*) is known to cause human infections and since the late 1990s, community-onset antibiotic resistant infections (methicillin resistant *S*. *aureus* (MRSA)) continue to cause significant infections in the United States. Skin and soft tissue infections (SSTIs) still account for the majority of these in the outpatient setting. Machine learning can predict the location-based risks for community-level *S*. *aureus* infections. Multi-year (2002–2016) electronic health records of children <19 years old with *S*. *aureus* infections were queried for patient level data for demographic, clinical, and laboratory information. Area level data (Block group) was abstracted from U.S. Census data. A machine learning ecological niche model, maximum entropy (MaxEnt), was applied to assess model performance of specific place-based factors (determined *a priori)* associated with *S*. *aureus* infections; analyses were structured to compare methicillin resistant (MRSA) against methicillin sensitive *S*. *aureus* (MSSA) infections. Differences in rates of MRSA and MSSA infections were determined by comparing those which occurred in the early phase (2002–2005) and those in the later phase (2006–2016). Multi-level modeling was applied to identify risks factors for *S*. *aureus* infections. Among 16,124 unique patients with community-onset MRSA and MSSA, majority occurred in the most densely populated neighborhoods of Atlanta’s metropolitan area. MaxEnt model performance showed the training AUC ranged from 0.771 to 0.824, while the testing AUC ranged from 0.769 to 0.839. Population density was the area variable which contributed the most in predicting *S*. *aureus* disease (stratified by CO-MRSA and CO-MSSA) across early and late periods. Race contributed more to CO-MRSA prediction models during the early and late periods than for CO-MSSA. Machine learning accurately predicts which densely populated areas are at highest and lowest risk for community-onset *S*. *aureus* infections over a 14-year time span.

## Introduction

*Staphylococcus aureus* (*S*. *aureus*) is a bacterium that is a part of normal human flora and also is a source of human infection. Approximately 30–40% of humans can be asymptomatic ‘carriers’ of *S*. *aureus* [1], and from the late 1990s until recently, community-onset antibiotic resistant *S*. *aureus* infections, also known as methicillin resistant *S*. *aureus* (CO-MRSA), have increased dramatically in causing both non-invasive and invasive infections [2].

Infections due to community-onset *S*. *aureus* (CO-*S*. *aureus*) appear to be increasing nationally and globally [3]. Skin and soft tissue infections (SSTIs) account for most community-onset infections due to both CO-MRSA and community-onset methicillin sensitive *S*. *aureus* (CO-MSSA) [4, 5]. Moreover, over the last decade, while community-onset SSTIs continue to occur at high rates, the etiology has proportionately shifted more to CO-MSSA than CO-MRSA [6, 7]. Risk factors for these community-onset infections include densely populated areas [8, 9] and populations which are socioeconomically disadvantaged [10]. Race and ethnic disparities exist for community-onset *S*. *aureus* infections, and risks associated with pediatric-related infections include daycare attendance, prior antibiotic use, family history of SSTIs, and public health insurance [10, 11].

However, the relationship between specific geographic location and risks tied to location for *S*. *aureus* infections has not been well characterized. Although several studies have explored socio-ecological risk factors for CO-MRSA [8, 12], the place-based associations between patients with CO-MRSA infections and identified risks have not been elucidated [9]. Moreover, the location-based associations tied to risk for staphylococcal infections at the community level among children have only been recently described for an urban community in the southeastern part of the United States [9].

Maximum entropy (MaxEnt) is a machine learning technique based on the principles of ecological niche modeling, which predicts the distribution of disease vectors and their possible disease transmissions using environmental and other relevant location-based risk factors [1, 13–16]. Although MaxEnt software [17] has been used in settings to predict human infection, e.g., leptospirosis, malaria, Dengue Fever, and influenza H7N9 [1, 13, 18, 19], there are no studies exploring its applicability to predict community-onset human infections in the U.S. In these reports, bioclimatic, environmental, and economic variables were selected *a priori* and then evaluated to determine their contribution to the transmission patterns. Using machine learning methods that factor in neighborhood level socio-ecological conditions to predict the *S*. *aureus* (‘species’) distribution has not been previously done. Moreover, the application of artificial intelligence (AI) to predict risks for *S*. *aureus* infections which originated in the community and includes place-based socio-ecological variables, has not been previously reported. This is the first study to use machine learning, based on species distribution niche modeling, to address the transmission of a bacterium for which humans are the natural host from 2002–2016, a period when CO-MRSA infections were occurring at epidemic rates.

## Material and methods

### Study population

Children treated for community-onset *S*. *aureus* infections from January 2002 through December 2016 were included in the data analyzed as previously described [9]. All patients were treated from a single pediatric healthcare system (Children’s Healthcare of Atlanta in Atlanta, Georgia, United States); this pediatric-dedicated healthcare system is the largest pediatric healthcare system in the state of Georgia and serves more than half a million children and adolescents annually [20].

#### Patient level data

**Fig 1:**
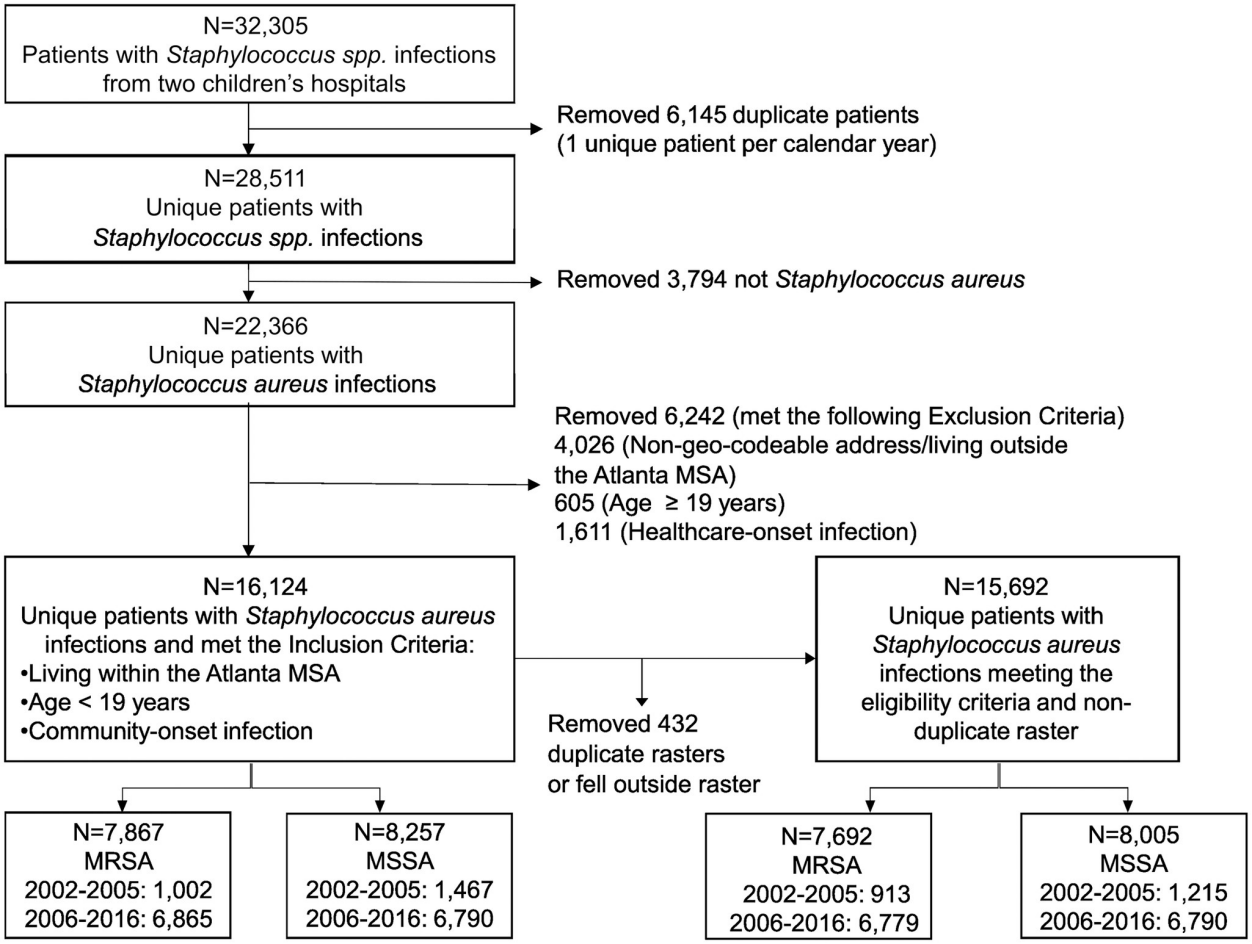
Enrollment schema.

Electronic medical records of patients (<19 years of age) seen at Children’s Healthcare of Atlanta with staphylococcal infections were obtained as previous described [21]. Specifically, Individual patient electronic health records were abstracted for demographic and clinical variables as outlined in Table 1. Enrollment scheme (Fig 1) summarizes the selection process: Briefly, each patient identified to have a positive Staphylococcus aureus culture was included for data abstraction for the following information: (1) sociodemographic characteristics (race ethnicity, age, gender, type of health insurance, postal residential address (street number and name, zip code), (2) clinical data (data of admission, date of discharge, final diagnosis and diagnosis codes based on International Classification of Diseases, Ninth and Tenth Revision Clinical Modification (ICD-9-CM and ICD-10-CM)) and (3) microbiology lab results (date of specimen collection and oxacillin susceptibility results). Each infection was categorized as community-onset infections (CO) or hospital-acquired (HA), where CO is defined as a positive *S*. *aureus* culture within 48 hours between healthcare visit date and date of specimen collection of positive *S*. *aureus* culture result (location or access point into initial healthcare was noted for any of the following areas: urgent care, emergency department, inpatient unit, ambulatory/outpatient primary or specialty clinic) [9] Furthermore, infections were classified as MRSA or MSSA based on oxacillin susceptibility results, where resistance to oxacillin was categorized as MRSA and susceptibility to oxacillin as MSSA. While we included all diagnoses in our enrollment of study participants, we did also determine which infections were considered SSTI based on ICD-9-CM and ICD-10-CM code, given that the majority of community onset staphylococcal disease are SSTIs.

**Table 1:** Population characteristics (patient level- and area level variables).

Characteristic Variable	Definition	Source
**Individual Level Data**		
Race	Black, White, Other (Asian, multi-races, and unknown races)	EMR*
Ethnicity	Hispanic, Non-Hispanic	EMR
Age	≤ 18 years (inclusive of 18 years)	EMR
Gender	Female, Male	EMR
Health Insurance	Public, Private, Both, Other (self-pay or both private and public types of health insurance)	EMR
*Staphylococcus aureus* culture	Microbiology culture result, based on resistance to Oxacillin (MRSA-methicillin resistant *S*. *aureus*; MSSA-methicillin sensitive *S*. *aureus*)	EMR
Skin or Soft Tissue Infection (SSTI)	ICD Diagnosis Code	EMR
Previous Hospitalization	Any hospital encounters within previous 12 months, or within any of the prior years of study period	EMR
Date of Admission, Date of Discharge, Date of Culture Collection for *S*. *aureus*	Date(s) associated with admission, discharge, or actual collection date of microbiology culture from source of infection which resulted in positive *S*. *aureus*	EMR
**Population Level Data**		
Pediatric Population	Percent < 18 years of age (not inclusive of 18 years)	ACS** 2010
Race—White	Percent of Population -White Race	ACS 2010
Race—Black	Percent of Population -Black Race	ACS 2010
Ethnicity	Percent of Population- Hispanic Ethnicity	ACS 2010
Poverty	Percent of Population, Living under Poverty Level in past 12 months	ACS 2013
Low Education	Percent of Population, ≥ 25 years of age with High School Diploma	ACS 2013
High Education	Percent of Population, ≥ 25 years of age with Bachelor’s Degree	ACS 2013
Household Crowding	Percent of Occupied Housing with > 1 Person per Room (Proxy for Household Crowding)	ACS 2013
Nursery School Enrollment	Percent of Population, ≥ 3 years, Enrolled in Nursery School	ACS 2013
Kindergarten Enrollment	Percent of Population, ≥ 3 years, Enrolled in Kindergarten	ACS 2013
School Distance	Mean value by Block Group of the Euclidean distance (feet) from each pixel to K-12 Schools. This was calculated using Zonal Statistics.	ARC***
Hospital distance	Mean value by Block Group of the Euclidean distance (feet) from each pixel to area hospitals. This was calculated using Zonal Statistics.	ARC
Daycare Centers distance	Mean value by Block Group of the Euclidean distance (feet) from each pixel to daycare centers. This was calculated using Zonal Statistics.	ARC
Population Density	Population Density (number of persons/mi^2^)	ACS

*EMR = Electronic Medical Record

**ACS = US Census, American Community Survey, data are provided based on the U.S. Census, block group geographic boundary. Depending on the year for the data on individual level variables, ACS data came from 2010 or 2013

***ARC = Atlanta Regional Commission Data; data are provided based on the block group boundaries, where applicable

To avoid counting repeated culture results of staphylococcal infections per unique patient, we counted only the first *S*. *aureus* culture and associated infection per unique patient per 12-month calendar year. The dataset for analyses also included only children who had a georeferenced residential address living within Atlanta’ Metropolitan Statistical Area (MSA); addresses were projected at the state plane level using assigned latitude and longitude coordinates and spatially joined to US Census block group boundaries (See Spatial Analyses Methods). Only patients who lived within the 20 counties of Atlanta’s MSA were included; this area is typically used for surveillance of different disease conditions by Georgia Department of Public Health [22]. If patients relocated within a calendar year, only the address associated with the first identified unique occurrence was georeferenced. This study was approved by the Institutional Review Boards of the respective academic institutions and healthcare system. Consent from parents or guardians and patients were waived by the Institutional Review Boards.

#### Area level data

Demographic, socio-economic, and socio-environmental characteristics of the population (i.e., neighborhood level risk factors) were obtained from the U.S. Census, American Community Survey (ACS), and the Atlanta Regional Commission as previously described [9] at the Block group level as outlined in Table 1. Demographic data (proportions within a specific block group) included race (black, white), ethnicity (Hispanic, non-Hispanic), poverty level, education level attained (high school diploma, bachelor’s degree), school enrollment (nursery school, kindergarten), population ≤18 years of age, and household crowding (housing unit with more than one person per room). Population density by U.S. Census Block Group was calculated for all block groups in the 20 counties of Atlanta’s metropolitan statistical area (MSA). Only block groups which represent areas where patients resided were included. In general, area variables during 2002–2005 were taken from the U.S. Census Bureau 2000, and area variables during 2006–2016 were obtained from U.S. Census Bureau 2010; for some variables, ACS data for the year closest to year of patient enrollment was used. For example, for population level variables, ACS 2010 was abstracted to align with patients enrolled in 2006, and for patients enrolled in 2016, ACS 2013 data was used. ACS 2013 data was used for the following area level variables: poverty, education attainment (high school diploma, bachelor’s degree), household crowding, nursery school enrollment, and daycare enrollment. This temporal division was created so that the two sets of decennial U.S. Census data variables, for 2000 and 2010, would more closely represent populations at the time of occurrence.

### Statistical analyses

Individual patient level and block group area level demographic variables based on previously reported risks [23] were selected for spatial-statistical analyses and machine learning model. Frequencies with percentages were used to describe the individual level variables for CO-MRSA and CO-MSSA. Chi-Square tests were performed to test association between individual level variables and *S*. *aureus* status (CO-MRSA and CO-MSSA). Area level variables for age, race, ethnicity, education attainment, poverty and housing risks were modeled as continuous and the odds ratio for CO-MRSA versus CO-MSSA were reported as either a 10%-unit change or, in distance variables (distance to school, daycare, or area hospital), the relevant distance unit changes. To consider both individual and area level variable effects, multilevel regression models were constructed, and SAS PROC GLIMMIX procedure was used to examine the multilevel variables’ effect on *S*. *aureus* status. Statistical analysis was performed using SAS 9.4 (SAS Institute, Cary, NC). All tests for significance were two-tailed, and a p-value of < 0.05 was considered significant.

### Spatial analyses

#### Georeferencing

Geocoding of data was performed using ArcGIS Pro, 2.9 (ESRI, Redlands). Coordinates were projected to Georgia West State Plane, NAD83, feet (EPSG: 2240). Relevant boundaries included block group and county boundaries within the 20 counties of Atlanta’s metropolitan statistical area (MSA). The primary unit of spatial analysis was the block group. A census block group represents about 400 households (1,200 individuals) and measures 1–2 square miles; block group is the smallest unit of census geography containing all the variables of interest. The Euclidean distance was calculated for daycare centers, K-12 schools, and children’s hospitals, and then a raster dataset with pixel values representing the distance from the pixel center to each point was generated. Since the other socio-economic variables were at the block group level, the mean distance per block group was calculated for each variable with a distance parameter (daycare, school, or hospital). Any issues with the modifiable areal unit problem (MAUP) were eliminated by having all the variables at the same spatial scale [24].

#### Maximum entropy models

We used MaxEnt software (v.3.4.4) to generate the maximum entropy distribution that satisfies the constraints determined by a set of relevant place-based socio-environmental variables [17]. To compare the potential risk of CO-MRSA and CO-MSSA within 20 counties of Atlanta’s MSA, four models were developed: two models (early and late period) for CO-MRSA occurrences and two (early and late) for CO-MSSA occurrences. The georeferenced occurrence locations for CO-MRSA and CO-MSSA from individual electronic healthcare records served as the model’s ecological niche or the designated ‘range of species’ [25]. The division between CO-MRSA and CO-MSSA was created to evaluate differences in predicted locations of the two different ‘habitat niches’. We used 14 reported area factors to serve as independent variables in the MaxEnt models; these were chosen based on risk factors we and others previously reported for CO-MRSA or CO-MSSA (Table 1) [26]. We limited the total number of variables to 14 to avoid collinearity and model overload. Vector data associated with variables of interest were converted to a raster with identical spatial resolution, pixel alignment and horizontal/vertical pixel dimensions using QGIS v.3.22 [27]. The spatial resolution was set to approximately 328 feet (100 meters) as this achieved a balance between providing sufficient resolution within each block group and acceptable model run times. We then used the Geospatial Data Abstraction Library Translate utility to conduct the necessary alignment and convert the files to ascii format [28]. Using the R package ENMeval (v2.0.3), the linear, quadratic, and hinge features were selected based on lowest AIC [29]. Infections occurring in the same raster were considered duplicate points and removed in each of the four models. Other settings were kept as default. The output format selected was logistic.

#### Mapping analyses

The dataset was divided into two time periods (2002–2005 and 2006–2016) as described above and U.S. Census 2000 block group boundaries were used for early period and U.S. Census 2010 block group boundaries were used for late period. The outputs of each of the four models (where only one infection occurring in each 100 m^2^ raster was included in each model) were mapped to visualize the potential occurrences of CO-MRSA and CO-MSSA in the early and late periods. The map values range from 0 to 1 with high values representing an increased probability of occurrence and zero representing no probability. To understand the spatio-temporal trends in CO-MRSA and CO-MSSA, raster algebra was performed on the results. For each period (early and late time span), the CO-MSSA model output was subtracted from the CO-MRSA output to show where each type *of S*. *aureus* was more likely to occur relative to the other.

#### Model assessment

We measured model performance by subsampling, where a portion of occurrence points are set aside for model evaluation. MaxEnt then determines how well the resulting niche model predicts these evaluation points. For each run, 75% of the occurrence points were randomly selected for model training and remaining 25% of points were used for model evaluation. Using two sets of points, MaxEnt generates receiver operating characteristic (ROC) plots which help determine the predictive capabilities of the MaxEnt model runs. Model accuracy, as measured by how close the area under the ROC (AUC) approached a value of one, is calculated internally in MaxEnt [30]. We also evaluated model performance by determining the predicted omission rate. This binomial test is based on actual omission and predicted omission areas to test the suitability of the model in prediction and then determine the significance of any differences detected between the omitted and ‘predicted omitted’ areas [17, 31]. The omission rate represents the number of test locations which fall outside an area predicted by the model.

To measure the relative importance of each socio-ecological variable in the four MaxEnt models, a jack-knife test was performed, which is an alternative way to look at which socio-ecological determinants are most important to the respective model(s) [32]. To compute these figures, MaxEnt runs iterations on each variable in isolation, and another set of model runs where each variable is excluded.

## Results

From 22,366 *S*. *aureus* infections (2002–2016), there were 16,124 unique occurrence points for CO-*S*. *aureus*: 7,867 CO-MRSA occurrences (1,002 for early period and 6,865 for late period) and 8,257 CO-MSSA occurrences (1,467 for early period and 6,790 for late period) (Fig 1). All occurrence points were within the 20 county MSA. Our analyses included 78% (2,609 block groups) of 3,335 block groups within the 20 county MSA.

### Population characteristics

When comparing CO-MRSA to CO-MSSA, significant differences were seen in the distribution of race, gender, age groups, types of health insurance, SSTI diagnosis, and prior hospitalizations for *S*. *aureus* infections (Table 2). In the early period (2002–2005), percentage of white children with CO-MSSA infections was higher compared to CO-MRSA infections, 65% and 49%, p <0.0001, respectively. The percentage of black children with CO-MSSA was lower than CO-MRSA, 21% and 39%, p <0.0001. Similar to the early period, white children during the late period had a higher percentage of CO-MSSA infections compared to CO-MRSA infections (56% and 44%, p <0.0001) whereas black children had a lower percentage of CO-MSSA infections compared to CO-MRSA infections (35% and 48%, p <0.0001). Among 1,558 Hispanic children with *S*. *aureus* infections,12% had CO-MRSA compared to 16% with CO-MSSA, p<0.0001. In both time periods, the percentage of younger aged children (≤3 years old) was highest among the age groups for CO-MRSA infections (46% in early period and 55% in late period), and older aged children (4–12-year-old) had a higher percentage of CO-MSSA (46% in early period and 40% in late period). In both time periods, public health insurance was also significantly higher with CO-MRSA (52% in early period and 60% in late period, p<0.0001) whereas private health insurance was associated with CO-MSSA only in early period (62% in early period and 43% in later period, p<0.0001). Skin and soft tissue infections (SSTIs) made up 75% of CO-MRSA infections, compared to 48% of CO-MSSA in the early period, p<0.0001. In the later period, SSTIs made up 37% of CO-MRSA infections and 25% of CO-MSSA infections, p<0.0001. Children with CO-MRSA infections had a lower percentage of prior hospitalizations (11% early period and 34% late period, p<0.0001) compared to those with CO-MSSA (28% early and 46% later period, p<0.0001).

**Table 2:** Patient level characteristics, early (2002–2005) and late (2006–2016) periods.

Patient Characteristic	CO-MRSA	Percent of CO-MRSA	CO-MSSA	Percent of CO-MSSA	p-value***
	(n = 7,867)		(n = 8,257)		
**Time period**					< .0001
2002–2005	1,002	13%	1467	18%	
2006–2016	6,865	87%	6790	82%	
**Race**					
2002–2005					< .0001
Black	386	39%	311	21%	
White	493	49%	950	65%	
Other*	123	12%	206	14%	
2006–2016					< .0001
Black	3,206	48%	2,259	35%	
White	2,938	44%	3,633	56%	
Other*	541	8%	654	10%	
**Ethnicity (Hispanic)**					
2002–2005	N/A	N/A	N/A	N/A	N/A
2006–2016	627	12%	931	16%	< .0001
**Age Group**					
2002–2005					< .0001
0–3 years	457	46%	472	32%	
4–12 years	331	33%	677	46%	
>12 years	214	21%	318	22%	
2006–2016					< .0001
0–3 years	3803	55%	2504	37%	
4–12 years	1967	29%	2715	40%	
>12 years	1095	16%	1571	23%	
**Gender (Female)**					
2002–2005	511	51%	638	43%	0.0002
2006–2016	3,459	50%	3,062	45%	< .0001
**Health Insurance**					
2002–2005					< .0001
Private	449	45%	914	62%	
Public	519	52%	510	35%	
Other**	34	3%	43	3%	
2006–2016					< .0001
Private	2,309	34%	2,926	43%	
Public	4,148	60%	3,571	53%	
Other**	408	6%	293	4%	
**Skin & Soft Tissue Infection Diagnosis**					
2002–2005	750	75%	704	48%	< .0001
2006–2016	2516	37%	1702	25%	< .0001
**Prior Hospitalization**					
2002–2005	113	11%	417	28%	< .0001
2006–2016	2341	34%	3133	46%	< .0001

*Other races include multi-races and unknown races

**Other types of health insurance include self-pay or both private and public types of health insurance

*** p<0.05 are statistically significant

#### Patient level risks for CO-MRSA

Black children are 57% more likely than white children to have a CO-MRSA infection (aOR 1.569, 95% CI: 1.460, 1.687). (Table 3) Females are 20% more likely than males to have CO-MRSA infections (aOR 1.200, 95% CI: 1.124–1.282). Children ≤3 years old are 81% more likely than those >12 years old to have CO-MRSA infections (aOR 1.810, 95% CI: 1.656–1.979). Children with public health insurance were 39% more likely than children with private health insurance to have CO-MRSA infection (aOR 1.263, 95% CI: 1.263, 1.521). Children with a prior hospitalization for staphylococcal infection were 32% less likely to have CO-MRSA infections (aOR 0.683, 95% CI: 0.623, 0.749). A clinical diagnosis of SSTI increases the risk of a child having a CO-MRSA infection by 75% (aOR 1.748, 95% CI: 1.628, 1.876).

**Table 3:** Patient-level risk factors for community-onset MRSA (CO-MRSA) compared to community-onset MSSA (CO-MSSA).

Risk Factor		Crude Odds Ratio (95% CI)	p-value*	Adjusted Odds Ratio (95% CI)	p-value*
**Patient Level**					
**Race**	White	Reference	-	Reference	-
	Black	1.867 (1.746, 1.997)	< .0001	1.569 (1.460, 1.687)	< .0001
	Other	1.031 (0.923, 1.152)	0.5842	0.745 (0.662, 0.839)	< .0001
**Gender**	Male	Reference	-	Reference	-
	Female	1.255 (1.179, 1.335)	< .0001	1.200 (1.124, 1.282)	0.0001
**Age Group**	>12 Years	Reference	-	Reference	-
	0–3 Years	2.066 (1.898, 2.248)	< .0001	1.810 (1.656, 1.979)	< .0001
	4–12 Years	0.978 (0.895, 1.068)	0.6154	0.971 (0.886, 1.065)	0.5341
**Health Insurance**	Private	Reference	-	Reference	-
	Public	1.592 (1.493, 1.698)	< .0001	1.386 (1.263, 1.521)	< .0001
	Other	1.832 (1.576, 2.128)	< .0001	1.032 (0.933, 1.141)	0.5397
**Prior Hospitalization**	No	Reference	-	Reference	-
	Yes	0.601 (0.564, 0.641)	< .0001	0.683(0.623, 0.749)	< .0001
**Skin & Soft Tissue Infection Diagnosis**	No	Reference	-	Reference	-
	Yes	1.726 (1.617, 1.843)	< .0001	1.748 (1.628, 1.876)	< .0001

*p<0.05 are statistically significant

#### Population level risks for CO-MRSA

Population density itself was significantly associated with *S*. *aureus* infection (p = 0.0012), but this variable was no longer significant in the adjusted model (p = 0.5864). (Table 4). For every 10% difference in race or ethnicity, there was a decrease in CO-MRSA risk for whites and Hispanics in the adjusted model (whites: aOR 0.917, CI: 0.875–0.962, p-value <0.0003; Hispanics: aOR 0.911, CI: 0.871–0.963, p-value <0.0001). While blacks also demonstrated an increased risk for CO-MRSA, this risk did not persist in the adjusted model (aOR 1.003, CI:0.958–1.050, p = 0.9054). Location and distance between patients’ georeferenced place of residence and nearest daycare center demonstrated a 3% increased risk for CO-MRSA with every 1,000 feet of distance between these two points in the adjusted model (aOR: 1.032, 95% CI: 1.012–1.052).

**Table 4:** Neighborhood-level risk factors for community-onset MRSA (CO-MRSA) compared to community-onset MSSA (CO-MSSA).

Risk Factor*	Crude Odds Ratio (95% CI)	p-value**	Adjusted Odds Ratio (95% CI)	p-value**
**Population Density (per 10,000/SQML)**	1.204 (1.076, 1.348)	0.0012	0.957 (0.819, 1.120)	0.5864
**Proportion Age under 18**	1.018 (0.964–1.076)	0.5180	0.907 (0.849–0.968)	0.0033
**Proportion White Race**	0.909 (0.900, 0.918)	< .0001	0.917 (0.875, 0.962)	0.0003
**Proportion Black Race**	1.101 (1.090, 1.112)	< .0001	1.003 (0.958, 1.050)	0.9054
**Proportion Hispanic**	0.962 (0.951–0.973)	< .0001	0.911 (0.871–0.953)	< .0001
**Proportion, High School Diploma**	0.975 (0.956–0.995)	0.0127	0.972 (0.960–0.985)	< .0001
**Proportion Bachelor’s Degree**	0.852 (0.831, 0.874)	< .0001	0.914(0.879,0.950)	< .0001
**Proportion Below Poverty Level**	1.139 (1.115, 1.164)	< .0001	1.009 (0.979, 1.040)	0.5636
**Proportion Enrolled in Nursery School**	0.818 (0.685, 0.976)	0.0256	0.985 (0.816, 1.190)	0.8783
**Proportion Enrolled in Kindergarten**	0.872 (0.706, 1.076)	0.2019	0.920 (0.737, 1.148)	0.4601
**Proportion of Housing Occupied with >1 person per room**	1.004 (1.003, 1.005)	< .0001	1.003 (1.001, 1.004)	< .0001
**Average distance to School (per 1,000 feet increase)**	0.981 (0.972, 0.990)	< .0001	0.985 (0.967, 1.003)	0.0932
**Average distance to Daycare (per 1,000 feet increase)**	0.982 (0.972, 0.991)	0.0001	1.032 (1.012, 1.052)	0.0016
**Average distance to Area Hospital (per 10,000 feet increase)**	0.988 (0.983, 0.994)	< .0001	0.998 (0.988, 1.008)	0.6390

*For rates on age, race, ethnicity, education attainment, poverty and housing risks, the odds (crude and adjusted, along with the 95% confidence interval) of a 10% increase in CO-MRSA compared to CO-MSSA are reported.

**p<0.05 are statistically significant

#### Patient and population level risks for CO-MRSA

The multilevel model (Table 5) demonstrated that individual level risk factors (race, gender, youngest age group, insurance, clinical diagnosis of SSTI, and prior hospitalization) remained significantly associated with CO-MRSA infections. In comparison to white children, black children remained more likely to have CO-MRSA (aOR 1.257,95% CI: 1.135, 1.392). Children less <3 years old had an 83% (aOR: 1.825, 95% CI: 1.659, 2.007) increase in risk of CO-MRSA infection after adjusting for both individual level and area level variables. Children with public health insurance were 20% more likely to have CO-MRSA infection compared to private health insurance (aOR: 1.204, 95% CI: 1.086, 1.335). A diagnosis of SSTI was also more likely to be associated with CO-MRSA compared to CO-MSSA (aOR 2.141, 95% CI: 1.977, 2.319). While many of the area level variables were no longer significant in the multi-level model, the aOR for average distance to day care, proportion with high school diploma, and proportion having a Bachelor’s degree remained significant.

**Table 5:** Multilevel model to assess risks for CO-MRSA compared to CO-MSSA.

Risk Variable*		Adjusted Odds Ratio (95% CI)	p-value**
Race	White	Reference	-
	Black	1.257(1.135, 1.392)	0.0001
	Others	0.738 (0.645, 0.843)	< .0001
Gender	Male	Reference	-
	Female	1.207(1.126, 1.294)	0.0001
Age Group	>12 Years	Reference	-
	0–3 Years	1.825 (1.659, 2.007)	< .0001
	4–12 Years	0.980 (0.889, 1.079)	0.6766
Health Insurance	Private	Reference	-
	Public	1.204 (1.086, 1.335)	< .0001
	Other	0.774 (0.687, 0.872)	0.0004
Prior Hospitalization	No	Reference	-
	Yes	0.739 (0.668, 0.816)	< .0001*
Skin & Soft Tissue Diagnosis	No	Reference	-
	Yes	2.141 (1.977, 2.319)	< .0001
Population Density per 10,000/SQML		0.881 (0.721, 1.078)	0.2183
Proportion Age under 18 Years		0.941 (0.868–1.021)	0.1434
Proportion White Race		0.948 (0.892–1.007)	0.0825
Proportion Black Race		1.005 (0.947, 1.068)	0.8614
Proportion Hispanic Ethnicity		0.948 (0.893–1.006)	0.0785
Proportion Having High School Diploma		0.924 (0.910–0.939)	< .0001
Proportion Having Bachelor’s Degree		0.950 (0.905–0.997)	0.0385
Proportion Below Poverty Level		1.027 (0.989, 1.067)	0.1707
Proportion Enrolled in Nursery School		0.971 (0.768, 1.227)	0.8045
Proportion Enrolled Kindergarten		0.852 (0.646, 1.124)	0.2576
Proportion Housing Occupied with >1 per room		1.001 (1.000, 1.003)	0.0691
Average distance to School (per 1,000 feet change)		0.984 (0.962, 1.007)	0.1670
Average distance to Daycare (per 1,000 feet change)		1.035 (1.010, 1.060)	0.0063
Average distance to Area Hospital (per 1,000 feet change)		1.004 (0.992, 1.017)	0.5154

*For rates on age, race, ethnicity, education attainment, poverty and housing risks, the odds (crude and adjusted, along with the 95% confidence interval) are reported as a 10% increase in CO-MRSA compared to CO-MSSA are reported.

** p<0.05 are statistically significant.

### Machine learning model performance

**Fig 2:**
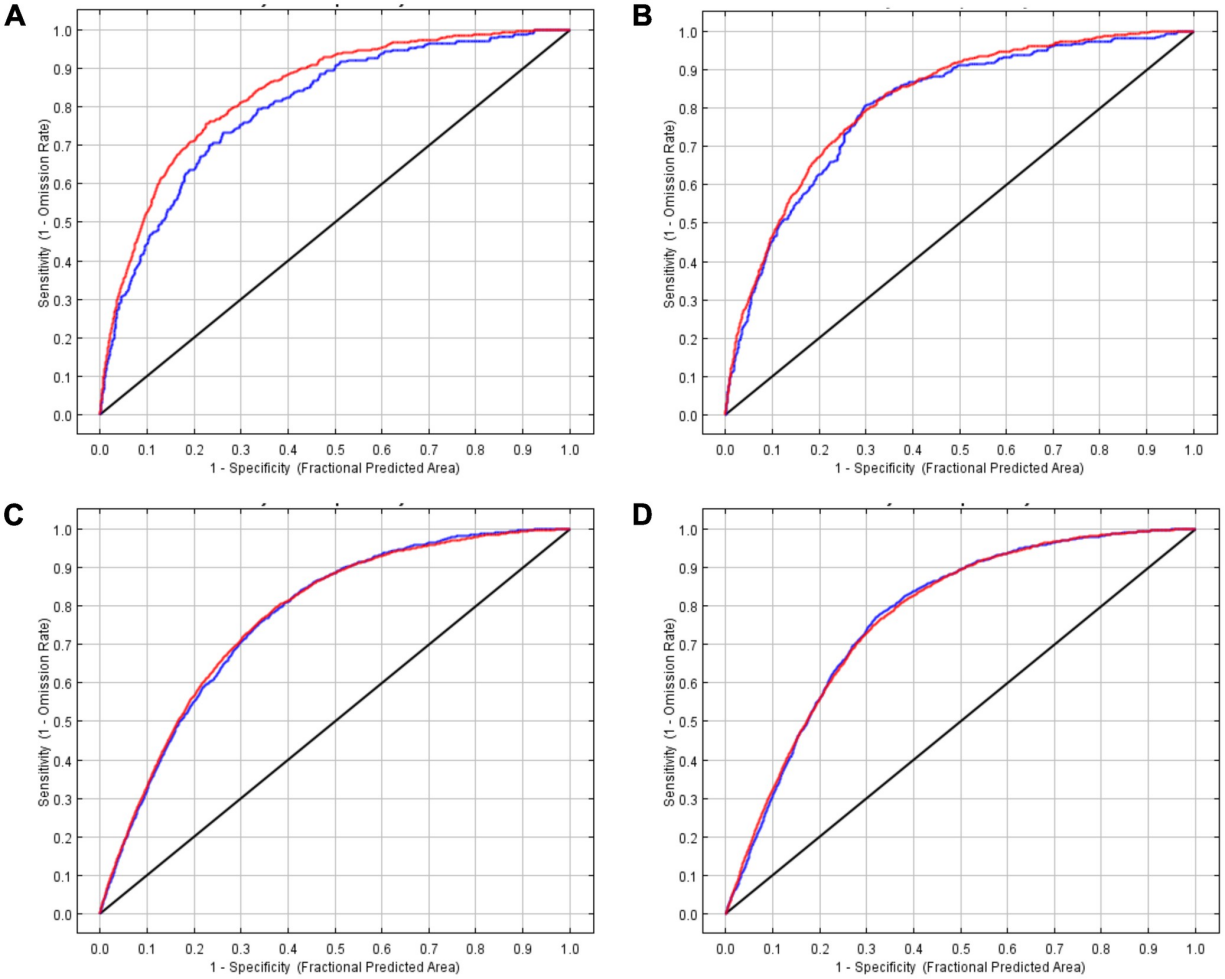
AUC curves for A) 2002–05 CO-MRSA, B) 2002–05 CO-MSSA, C) 2006–16 CO-MRSA, and D) 2006–16 CO-MSSA training and evaluation sites. Black line represents random prediction. Red line represents training data. Blue line represents test data.

We included 15,094 unique *S*. *aureus* occurrence points. 1,030 points from the 16,124 unique patients were excluded from the analyses because points (1) fell outside the rasterized study areas or (2) there was more than one occurrence within a raster (see Fig 1 for details of excluded points by time period and type of *S*. *aureus*). Receiver Operating Characteristic (ROC) along with the associated Area Under the Curve (AUC) for both training and evaluating points are shown in Fig 2. All four model runs had similar very high AUCs for both training and evaluation data. The training AUCs ranged from 0.771 to 0.837. For the evaluation points, the AUC values were far closer to 1 than 0.5, ranging from 0.769 to 0.804, which demonstrates a high predictive power. S1 Fig shows how evaluating and training omission and predicted areas vary with the choice of cumulative threshold. For each run, the omission on training and evaluating samples are a very good match to the predicted omission rate; hence, demonstrating the models are good at predicting occurrences for both CO-MRSA and CO-MSSA.

### Predicting areas of community-onset MRSA and MSSA risks between early and late periods

**Fig 3:**
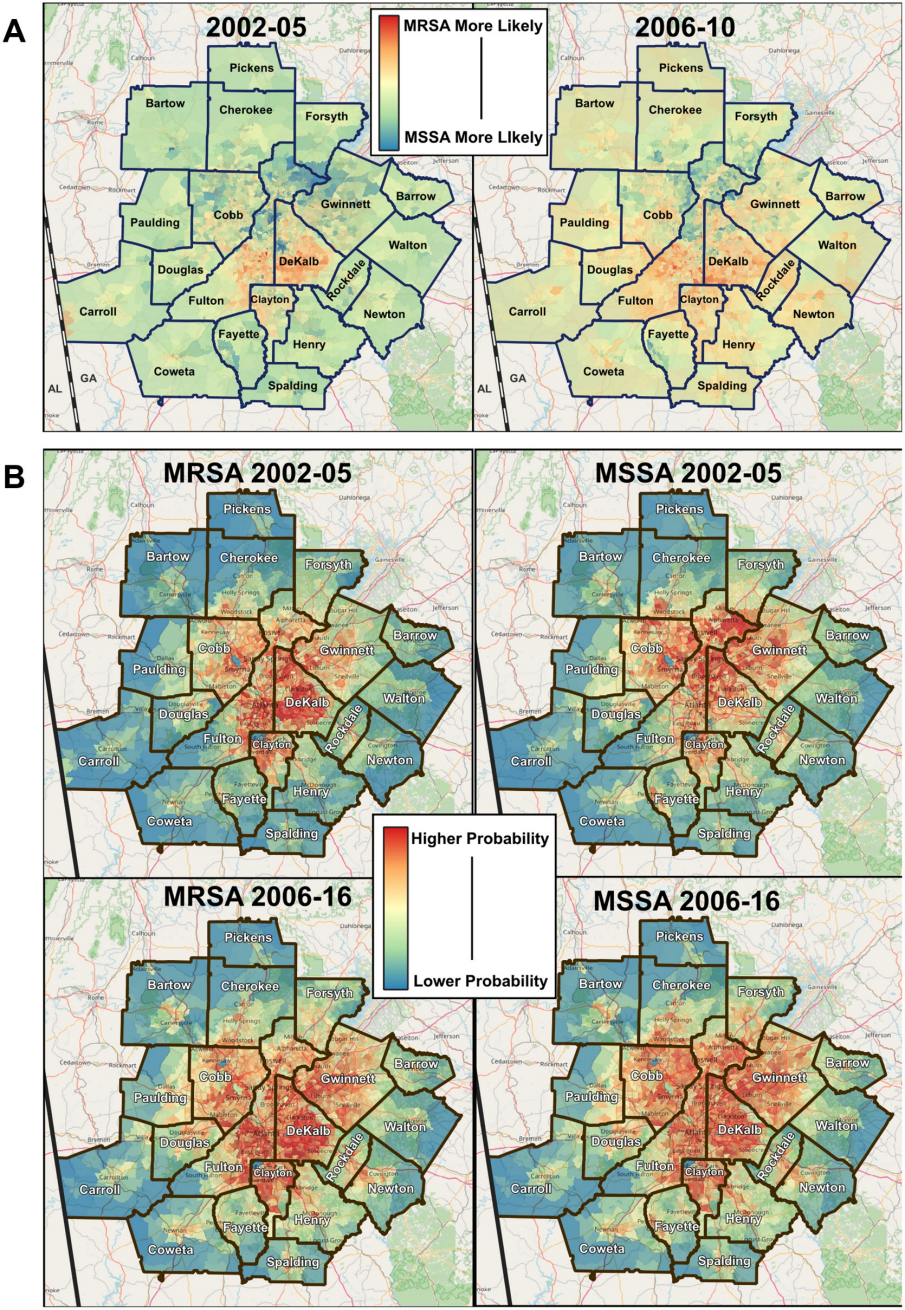
A) Comparing likely areas of CO-MRSA and CO-MSSA between early (2002–2005) and later (2006–2016) time periods. B) Predicting areas of CO-MRSA/MSSA risks between early and late periods.

The probability map of CO-MRSA and CO-MSSA for the MSA 20 counties, based on MaxEnt models is shown in Fig 3. The degree and areas of highest probability for both CO-MRSA and CO-MSSA differed between early (2002–2005) and later (2006–2016) model runs. Overall, the CO-MRSA occurrences in the early period remained in the same locations as the later period; the later period also included additional areas in the southern counties of the MSA not seen in the earlier period. In the 2002–2005 models, there is a higher probability of CO-MRSA occurrence in central Fulton County, southern Dekalb County and far western Carrol County. (These areas of Fulton and Dekalb counties are also the most densely populated counties for the state of Georgia.). In contrast, CO-MSSA occurrences were predicted to involve a larger geographic area, which expanded to northern Fulton County, portions of southern Forsythe, Gwinnett, and Cobb counties, similar to the population density in 2000 (S2 Fig). In the 2006–2016 model, areas predicted to have higher occurrences of *S*. *aureus* (both CO-MRSA and CO-MSSA) were larger than the areas seen in the earlier period (2002–2005). Specifically, CO-MRSA occurrences in 2006–2016 period were predicted to expand into a larger portion of eastern Douglas, southern Fulton, southern DeKalb, southern Clayton, western Newton, and central Spalding, Walton, and Henry counties. Henry and Newtown were counties not found in the earlier period (2002–2005); Rockdale County was predicted to occur in both early and late periods. However, there were also overlapping areas of ‘highest probability’ for both CO-MRSA and CO-MSSA in the 2006–2016 models; this is seen mostly in Fulton, Dekalb, Gwinnett and Cobb counties, which is similar to the population density in 2010 (S2 Fig). Analyses also predicted niches whereby areas of ‘highest probability’ for CO- MSSA in the early 2002–2005 model became areas of ‘highest probability for CO-MRSA in 2006–2016, e.g., segments of Barrow, Rockdale, and Walton counties.

In general, a shift to the south was seen for CO-MRSA and to a lesser extent, for CO-MSSA in 2006–2016 period. The CO-MSSA ‘niche’ in the later period involved more intensely the eastern and southern areas of Atlanta’s MSA 20 counties (especially Gwinnett and DeKalb counties), compared to CO-MRSA.

### Machine learning model predicts risk contributed by area variables

Variables included in the MaxEnt prediction models are shown in Table 6, along with the respective percent contribution and permutation importance. Areas of CO-MRSA occurrence were largely predicted by population density (80.2% in 2002–2005, and 79.9% in 2006–2016). In the prediction model for CO-MRSA, black race contributed the second highest (4.7%) in the 2002–2005 prediction model, whereas white race contributed the second highest (8.6%) in the 2006–2016 model. The third most contributing variable was average distance to area hospital (3.7%) and average distance to day care (3.8%) for the 2002–2005 and 2006–2016 prediction models, respectively. Areas of CO-MSSA were largely predicted by population density (84.8% in 2002–2005, and 92.2% in 2006–2016). In the 2002–2005 CO-MSSA prediction model, the second most contributing variable was proportion having a Bachelor’s degree (4.7%). Similar to the 2006–2016 CO-MRSA model, white race was the second most contributing variable in the 2006–2016 CO-MSSA model (2.1%). The third most contributing variable for both the 2002–2005 and 2006–2016 CO-MSSA prediction models was average distance to area hospital (3.6% and 2%, respectively).

**Table 6:** Percent contribution of variables for 2002–2005 CO-MRSA, 2002–2005 CO-MSSA, 2006–16 CO-MRSA, and 2006–16 CO-MSSA.

Variable	MRSA 02–05		MSSA 02–05		MRSA 06–16		MSSA 06–16	
	Percent Contribution	Permutation Importance	Percent Contribution	Permutation Importance	Percent Contribution	Permutation Importance	Percent Contribution	Permutation Importance
Population Density	80.2	84.1	84.8	72.4	79.9	89.3	92.2	92
Proportion Age under 18 Years	0.3	0.7	0.9	2.2	0.4	0.6	1.1	1.6
Proportion White Race	3.2	1.9	0	0.3	8.6	0.3	2.1	0.1
Proportion Black Race	4.7	2.2	0.9	2.2	3.2	1	0.6	0
Proportion Hispanic Ethnicity	1.1	0.7	0.9	0.6	0.2	0	0.1	0.4
Proportion with High School Diploma	0.1	0.6	0.1	0.4	0.3	0	0.1	0
Proportion with Bachelor’s Degree	0.4	1.9	4.7	5.9	0.1	0.5	0.1	0.2
Proportion Below Poverty Level	0.3	0.4	0.8	0.5	0.5	0	0	0
Proportion in Nursery School	0	0.7	0.2	0.2	0	0.1	0.2	0
Proportion in Kindergarten	0.9	0.9	0.6	0	0.1	0	0	0
Proportion Housing Occupied with >1 per room	0	0	0.9	1	0.1	0	0	0
Average distance to School	1.7	0.7	1.4	0.9	1.5	0	1.2	0
Average distance to Day Care	3.5	0.1	0.3	7.5	3.8	0	0.2	1.3
Average distance to Area Hospital	3.7	5	3.6	5.9	1.3	8.3	2	4.4

**Fig 4:**
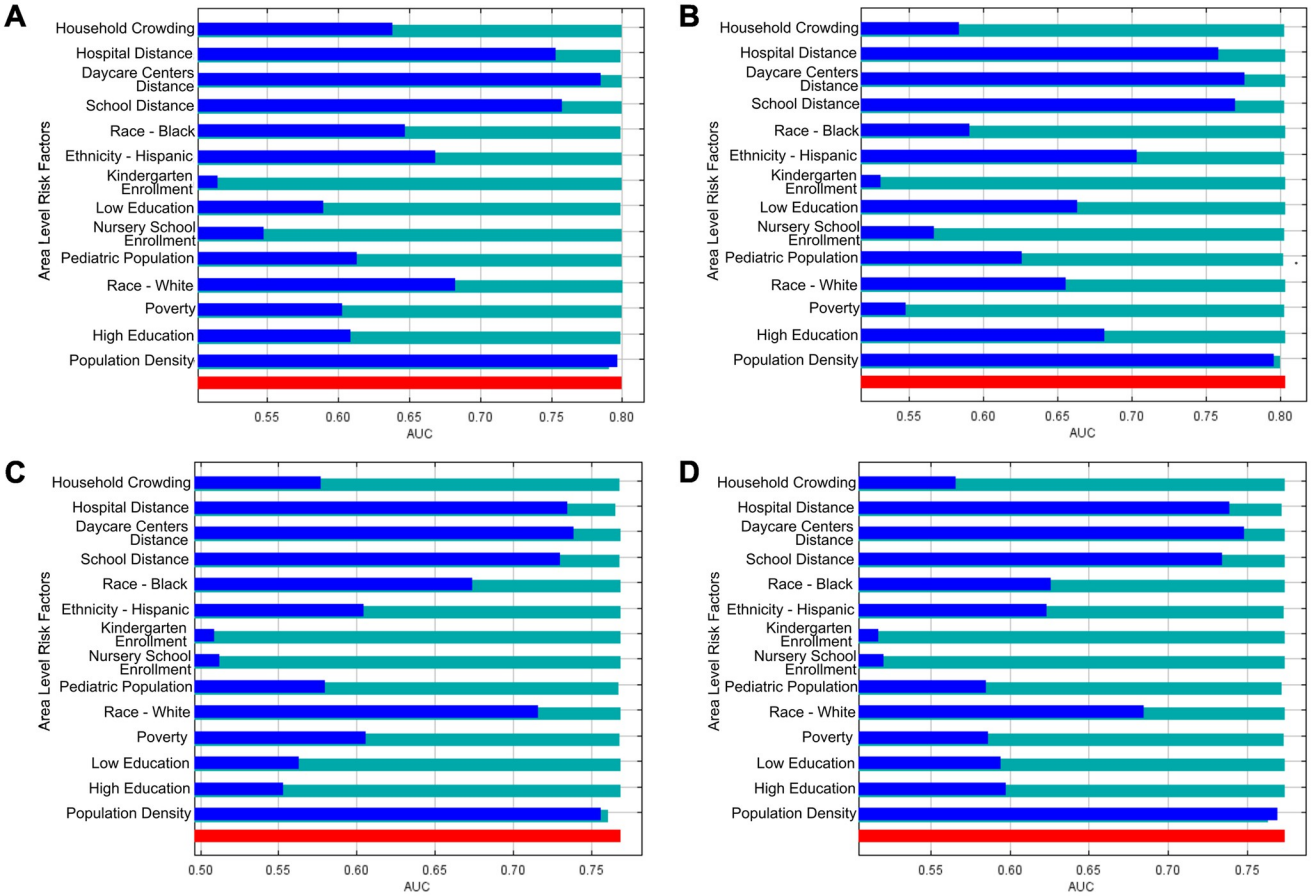
Jackknife of AUC for A) 2002–2005 CO-MRSA, B) 2002–2005 CO-MSSA, C) 2006–16 CO-MRSA, and D) 2006–16 CO-MSSA. Green bar is without variable. Blue bar is with only variable. Red bar is with all variables.

Applying the jackknife technique (Fig 4), each model performs nearly as well with population density in isolation, as with all the other variables. The three distance variables (distances to hospital, daycare, and school) were also important in predicting potential CO-MRSA and CO-MSSA niches. No more precision in predictive power is gained by including variables such as age or enrollment in schools. All model runs perform worse when population density variable is not included; removal of other variables did not significantly affect overall model performances.

## Discussion

We used MaxEnt modeling to predict what place-based variables contributed to the spread or transmission of both CO-MRSA and CO-MSSA in a major urban area in southeastern U.S. Our study indicated that densely populated areas was the single largest predictive variable for CO-MRSA and CO-MSSA occurrences. We also ascertained that specific background variables were important in determining the probability of CO*-S*. *aureus* occurrences, including location (and relative distance) to area pediatric hospitals and daycare centers. The distribution of race and ethnicity within a given geographic location also influenced the predictability of CO-MRSA occurrence but its contribution was far less than what might have been expected. Collectively, these findings demonstrate the importance of location-based socio-ecological variables in occurrence of *S*. *aureus* infections, which is different than simply stating how social determinant variables contribute to the health disparities seen with *S*. *aureus* infections. The geographic location plays an essential role in predicting risk for CO-*S*. *aureus* infections. While predictions from our machine learning application support what we reported in previous spatial analyses [9], our MaxEnt model informs us that population density among the 14 variables assessed was by far the single most important predictor of occurrence of this type of infection in children; we also can more accurately ascertain which particular locations are at higher or lower risk for these infections. Where previously, we and others also identified various social determinants that appear to increase risk for CO-MRSA infections, MaxEnt’s niche models are able to extend these analyses to examine location-based factors and degree of predictive power from these factors.

Since population density was the most important predictor of location-based CO-MRSA and CO-MSSA occurrence, other factors which affect or are affected by population density, not surprisingly, also contributed to a lesser degree. However, these other predictive factors changed between the early and late periods and also differed between CO-MRSA and CO-MSSA. For example, race (proportion white or proportion black) was the second most contributing factor in the MaxEnt models, except for the early period CO-MSSA model. In our prediction models, race contributed more in predicting CO-MRSA outcomes than CO-MSSA. We found that the predictive contribution of white race for CO-MRSA and CO-MSSA in an area increased from early to later period, not seen with black rates. This output from our machine learning model could reflect the changing demographics overall within the 20-county Atlanta MSA and demographics of children with CO-*S*. *aureus* infections.

Disparities across race and age with MRSA infections (including community and hospital acquired) have been longstanding in both adults and children. We recently reported for this same catchment area, black race was no longer a significant risk for MRSA SSTIs in either adults or children after adjusting for household level crowding [33]. While these findings previously reported are consistent with this study’s findings, our study is able to reliably predict the specific locations where there is the highest probability of CO-MRSA or CO-MSSA occurrence based on geographic locale and related risks tied to these locations, including the locations of area daycare centers and distribution of race and ethnic groups. Day care attendance has been an established risk factor for many childhood infections, including *S*. *aureus*, and access to these pediatric congregate care settings seems to be important in predicting risk for infection; the demand for numbers and types of daycare settings may be driven by the population density of an area. Household crowding has previously been identified as a risk factor for CO-*S*. *aureus* infections. In our MaxEnt models, household crowding contributed < 1% in the models. Duplicates of location (for example, 2 patients living within the same ‘raster’ location were counted as single occurrence) were removed in the MaxEnt models to ensure no bias was introduced, which may have deemphasized the contribution household crowding plays in CO-*S*. *aureus* infections.

While block groups with high population density also generally have higher poverty rates and lower education attainment rates, these two variables did not majorly contribute to the MaxEnt predictive models of CO-*S*. *aureus* occurrence. Comparatively, in our multilevel model, which did not factor in location of occurrence, we identified public health insurance (proxy for poverty) as a significant risk for CO-MRSA; however, we did not find increased rates of living below poverty level or increased rates of high school diploma as significantly associated with CO-MRSA infections. When we add in location-based variables into our MaxEnt model, we are able to refine these risk variables and take into consideration neighborhood influences (i.e., place-based factors) to better understand to what extent they contribute to probability of CO-MRSA occurrence in that area.

The study has the following limitations: First, the study selected 14 variables to predict the potential distribution of CO-MRSA/CO-MSSA infection; there may be other variables which are location based and are part of the ‘built environment’, e.g., transportation access, quality and types of housing that were not included but may impact predictive models; data for these variables are not as complete as the variables selected, and so were not included. (In future studies, we may rerun the MaxEnt models with replacement of those variables not found to contribute in current models with these ‘access’ variables.) Second, we could not differentiate in our analyses patients who self-identified beyond black, white, Asian for race, nor could we assign any particular Latinx sub category; we used health systems’ category assignment of ‘Hispanic’ as the only ethnic group. To better understand the risk of race and how race and ethnicity impact health outcomes (CO-*S*. *aureus* infections), the neighborhood level race factors need to be further elucidated. For example, in many urban areas, communities have cultural tie-ins bound by residential boundaries, so that a community with disproportionately higher rates of blacks actually reflect an ethnically diverse population of African Americans or first and second generations of Africans, Afro-Caribbeans, or Afro-Latinos; the cultural influences of these diverse racial communities on healthcare may vary based on the ethnocentricity of a community, which is not captured in the current predictive models for infection. Additionally, often cultural geographic boundaries differ from boundaries defined by census tract or block group. Comparing our studies’ findings to another setting where there are multiple healthcare systems for children may reveal whether or not distance to pediatric care facilities remains a significant contributor to prediction models for CO-MRSA and CO-MSSA infections. The generalizability of our findings may be limited to geographic areas similar to the area we included in this study—urban setting, in the southeastern U.S. However, a major strength of this study is the large georeferenced dataset, which includes a timespan of more than 10 years. Furthermore, our hospital system is inclusive of pediatric hospitalizations that likely accounts for >90% of those admissions for hospital management of this condition.

## Conclusions

Our MaxEnt models are able to generate accurate maps which predict areas with high to low probability of having CO-MRSA or CO-MSSA occurrence. This application of machine learning provides more value than simple spatial analyses (e.g., cluster or hot/cold spot analyses), because it will help local communities determine primary and secondary prevention strategies based on the local conditions relevant to the patients who live in the area. Geographical probability distribution of infections using an ecological niche model has the capability to identify those location-based risks with high sensitivity, using the resulted AUC. Moreover, MaxEnt modeling for CO-*S*. *aureus* infections can determine to what degree these location-based risks contributed to the probability distribution. As communities change, such as in racial and ethnic distribution, these models can be used to predict future areas of CO-MRSA and CO-MSSA occurrences based on how the relevant area variables, factoring the changes which are ongoing in real-time. This can assist in planning how resources are allocated to areas, based on projections of where occurrences of CO-MRSA or CO-MSSA are higher.

Predicted risk areas based on the ecological niche model distribution of CO-MRSA occurrences highlights to local public health agencies how and to what extent more frequent surveillance is necessary in order to prevent *S*. *aureus* outbreaks from occurring. As the population in the southern portion of Atlanta’s MSA grows, increasing surveillance and monitoring of these areas for *S*. *aureus* infections seems prudent, given the infection dynamic changes seen for these antibiotic resistant bacteria. Since the predictive models are built on geo-referenced socioecological characteristics, the resulting probability distribution will be based on ‘real’ world dynamics relevant to the local area. With the increase in antimicrobial resistance in CO-MSSA and steady numbers of CO-MRSA, it is imperative that hospitals and communities identify risk areas for *S*. *aureus* outbreaks and respond quickly and effectively to prevent transmission spread before they move into an epidemic status. This approach has utility beyond the scope of CO-MRSA and given the recent ongoing pandemic due to a novel coronavirus, SARS CoV-2, a data-driven ecological niche machine learning model seems imperative and needed more than ever.

## Supporting information

S1 Fig Omission vs. Predicted Area for A) 2002–2005 CO-MRSA, B) 2002–2005 CO-MSSA, C) 2006–16 CO-MRSA, and D) 2006–16 CO-MSSA. Red line represents fraction of background predicted. Blue line represents omission on training samples. Light blue line represents omission on test samples. Black line represents predicted omission.
(JPG)

S2 Fig Population densities by US Census 2000 and 2010 for Atlanta metropolitan statistical area (MSA). (JPG)

S1 Table Individual and area variables. Anonymized and limited dataset of patients enrolled in the study, stratified by the type of *Staphylococcus aureus* infection (CO-MRSA and CO-MSSA) and date (year) of infection.
(XLSX)

S2 Table Spatial tables. Dataset of species (MRSA v. MSSA), stratified by early and late periods, along with associated location coordinates (latitude and longitude) used to create MaxEnt maps.
(CSV)
