# Supplementary figures and images for: Machine learning to predict risk for community-onset Staphylococcus aureus infections in children living in southeastern United States
Source: PLoS One. 2023 Sep 1;18(9):e0290375. doi: 10.1371/journal.pone.0290375 (PMC10473480; doi:10.1371/journal.pone.0290375)

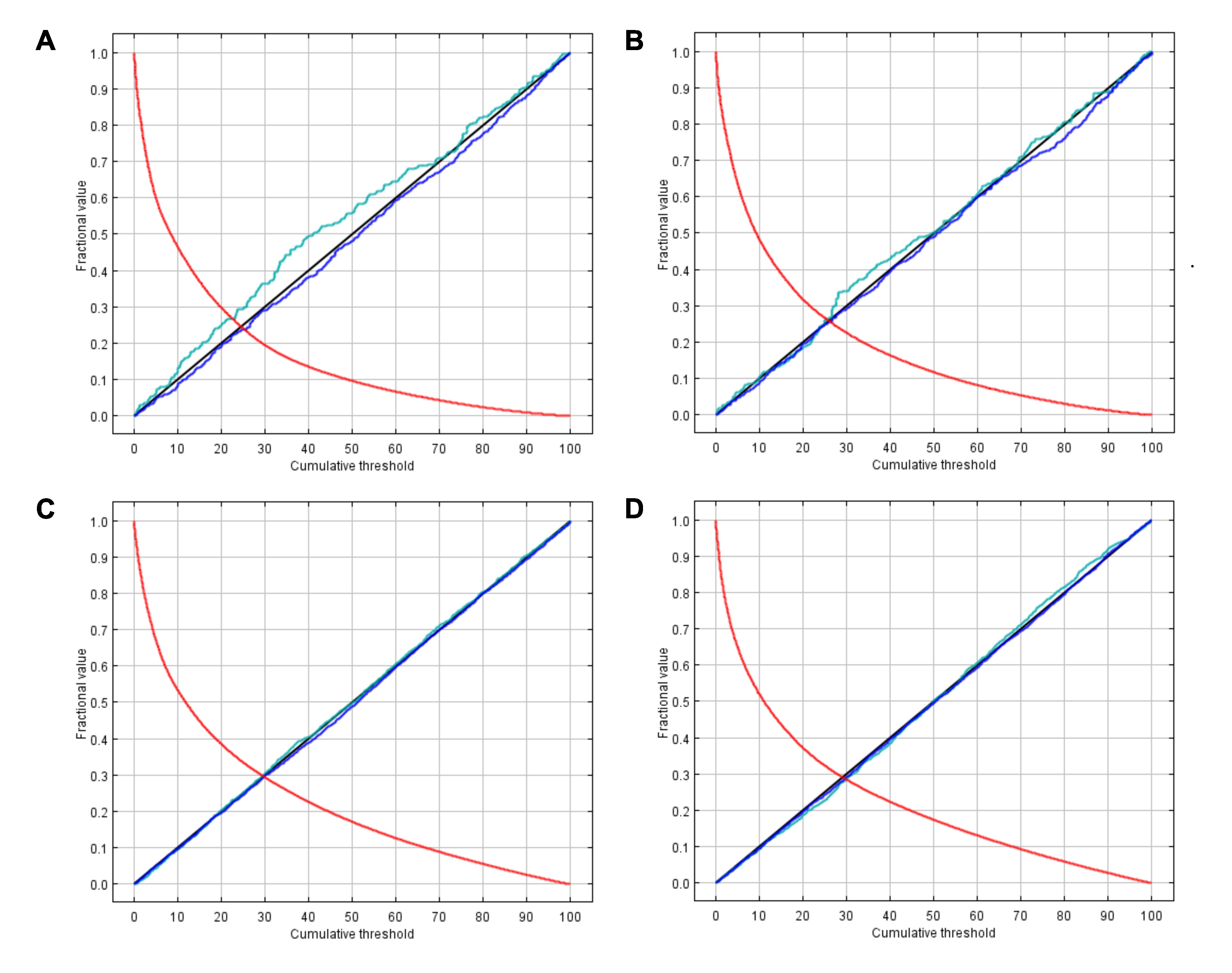

Supplement: S1 Fig — Red line represents fraction of background predicted. Blue line represents omission on training samples. Light blue line represents omission on test samples. Black line represents predicted omission. (JPG) [file pone.0290375.s001.jpg]

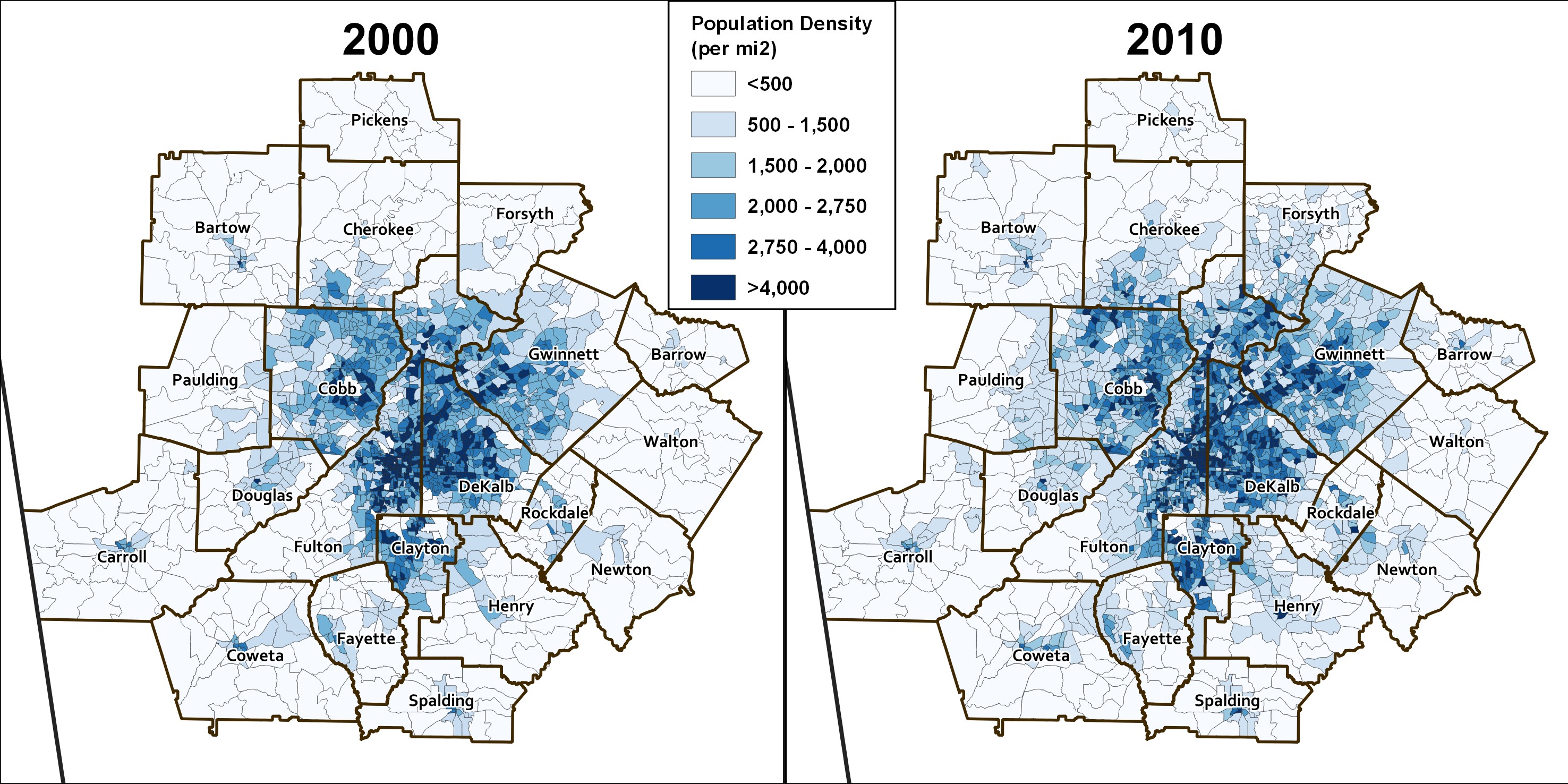

Supplement: S2 Fig — (JPG) [file pone.0290375.s002.jpg]
